# Chromophobe renal cell carcinoma: A case report and literature review

**DOI:** 10.1016/j.amsu.2021.102643

**Published:** 2021-07-28

**Authors:** Ahmed Ballati, Zakaria Essaidi, Saad Rifki El Jai, Amal Hajri, Driss Errguibi, Rachid Boufettal, Farid Chehab

**Affiliations:** Departement of General Surgery, University Hospital Centre Ibn Rochd, Faculty of Medecine and Pharmacy, Hassan II University, 9,Gascogne Street, Casablanca, Morocco

**Keywords:** Chromophobe renal cell carcinoma, Surgery, Partial nephrectomy

## Abstract

**Introduction:**

Chromophobe renal cell carcinoma, a distinct subtype of renal cell carcinoma (RCC) with characteristic light microscopic, histochemical, and ultrastructural features, typically has a favorable clinical course.

**Presentation of case:**

A 45-year-old femele presented with abdominal pain. A physical examination found a palpable mass in the left upper quadrant of the abdomen. A CT scan of the abdomen showed a heterogeneously enhancing mass, with necrosis and calcifications contents betwen the liver and the right kidney. she underwent surgical resection. Partial nephrectomy was performed. Pathological diagnosis was Chromophobe renal cell carcinoma.

**Discussion and conclusion:**

Chromophobe RCC is a rare variety of kidney neoplasm that has recently been better characterized from a molecular and genetic perspective. Overall, it is considered to have a better prognosis, and is associated with earlier stage tumors and longer overall survival compared with clear cell RCC.

## Highlights

• The occurrence Chromophobe renal cell carcinoma of are extremely rare.
• Complete surgical resection is the only potential curative treatment for chromophobe cell carcinoma.
• Overall, it is considered to have a better prognosis, and is associated with earlier stage tumors and longer overall survival compared with clear cell Renal Cell Carcinoma.

## 1 Introduction

Chromophobe renal cell carcinoma, a distinct subtype of renal cell carcinoma (RCC) with characteristic light microscopic, histochemical, and ultrastructural features, typically has a favorable clinical course, with only a small proportion of patients developing recurrence or metastasis or dying from disease [1]. This work has been reported in line with the SCARE criteria [2].

## 2 Case presentation

**Fig. 1:**
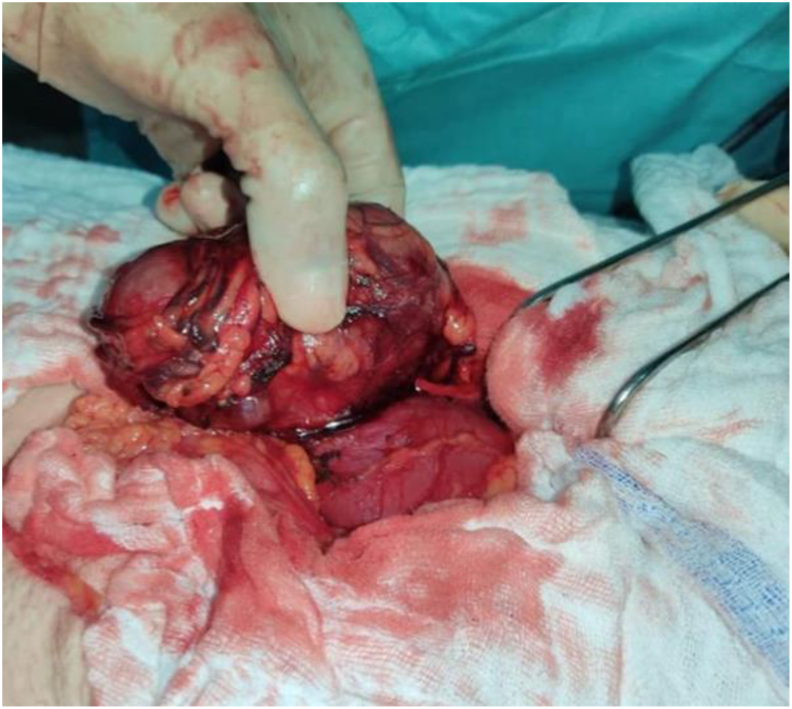
Intraoperative photograph showing the presence of a 6 cm hard mass in the upper pole of the right kidney.

**Fig. 2:**
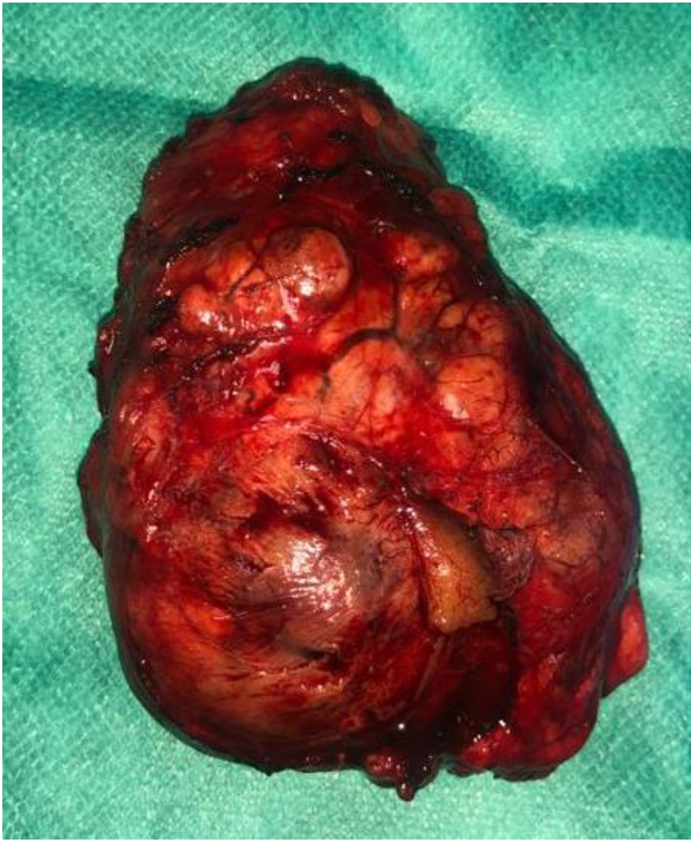
Specimen.

A 45-year-old femele not known to have any medical illness presented with abdominal pain. A physical examination found a palpable mass in the left upper quadrant of the abdomen. A CT scan of the abdomen showed a 63 × 21 × 60 mm heterogeneously enhancing mass, with necrosis and calcifications contents betwen the liver and the right kidney. she underwent surgical resection under general anesthesia using laparotomy, A pinkish white hard mass approximately 6 × 6 cm was seen in the upper pole of the right kidney (Fig. 1), no evidence of metastases or enlarged lymph nodes was found at abdominal exploration, Partial nephrectomy was performed. specimen showed a tumor at the upper pole, 67 × 65 × 51 mm with a beige cut surface (Fig. 2). Pathological diagnosis was Chromophobe renal cell carcinoma. Tumor cells were CK7 (+), CKAEI (+), CD10 (+), CD56 (−), PS100 (−). Histopathologial advancement was: pT1b.

## 3 Discussion

Renal cell carcinoma is heterogeneous disease which is caused by different histological variants, The most common subtype of renal cell carcinoma is clear cell accounting 75 %, papillary follows about 10 %, chromophobe 5 % and undifferentiated 10 % of all cases. Chromophobe renal cell carcinoma, a distinct subtype of renal cell carcinoma (RCC) with characteristic light microscopic, histochemical, and ultrastructural features [3].Several risk factors have been identified that are potentially responsible for the increasing number of newly-diagnosed renal cell carcinomas, among them smoking, hypertension, age, and male gender [4].Renal cell carcinoma can be diagnosed based on clinical presentation, Common presenting complaints and signs included: abdominal pain, hematuria, abdominal mass, and other symptoms, including decreasing renal function, proteinuria, and pain from metastatic site, they can be asymptomatic and are discovered while worked-up for other medical problems [5].Contrast-enhanced computed tomography (CT) detects 90 % of renal masses, identifies benign and pathologic features, and evalu¬ates surrounding anatomy to detect lymphadenopathy or an associated thrombus, For incompletely characterized masses or contraindica¬tions to CT, magnetic resonance imaging with and without intravenous contrast is recommended [6].The primary choice of the treatment of any stage of RCC is surgical excision, partial nephrectomy has emerged as the widely recommended treatment for small renal tumors, while Partial nephrectomy was initially reserved for cases with a contraindication to radical nephrectomy, such as solitary kidney, chronic kidney disease and multi-focal or bilateral tumors, it has now become the surgical gold standard for all small renal tumors when technically feasible, The advantage of nephron-sparing surgery lies in preservation of parenchyma and hence renal function [7].Studies that investigate the results of systemic therapies in patients diagnosed with metastatic Chromophobe RCC disease suggest that sunitinib have an advantage compare to everolimus without being statistically significant, and sunitinib seems to be superior than sorafenib, but the optimum therapy for Chromophobe RCC is still missing [8].Several large studies have indicated that prognosis of chromophobe RCC is much better than that of clear cell RCC and papillary RCC. Most chromophobe RCC have a favorable outcome and low risk of metastasis, but there is evidence that chromophobe RCC have a predisposition to metastasise into the liver [1,9].

## 4 Conclusion

Chromophobe RCC is a rare variety of kidney neoplasm that has recently been better characterized from a molecular and genetic perspective. Overall, it is considered to have a better prognosis, and is associated with earlier stage tumors and longer overall survival compared with clear cell RCC [9]. we report a case of a Chromophobe renal cell carcinoma, in whom a partial nephrectomy was performed.

## Provenance and peer review

Not commissioned, externally peer-reviewed.

## Funding

The authors declared that this study has received no financial support.

## Ethical approval

I declare on my honor that the ethical approval has been exempted by my establishment.

## Sources of funding

None.

## Registration of research studies

Not applicable.

## Consent

Written informed consent for publication of their clinical details and/or clinical images was obtained from the patient.

## Author contribution

Ahmed Ballati: Corresponding author writing the paper.

Zakaria Essaidi: writing the paper.

Saad Rifki El Jai: correction of the paper.

Amal Hajri: writing the paper.

Driss Errguibi: study concept.

Rachid Boufettal: study concept.

Farid Chehab: correction of the paper.

## Guarantor

DOCTEUR AHMED BALLATI.

## Declaration of competing interest

The authors declare having no conflicts of interest for this article.
